# Missing Fundamental Nursing Care: What’s the Extent of Missed Oral Care? A Cross-Sectional Study

**DOI:** 10.3390/nursrep14040305

**Published:** 2024-12-23

**Authors:** Chiara Gallione, Erika Bassi, Ines Basso, Chiara Airoldi, Michela Barisone, Antonella Molon, Gerardo Di Nardo, Cristina Torgano, Alberto Dal Molin

**Affiliations:** 1 Department of Translational Medicine, University of Piemonte Orientale, Via P. Solaroli, 17, 28100 Novara, Italy; ines.basso@uniupo.it (I.B.); chiara.airoldi@uniupo.it (C.A.); michela.barisone@uniupo.it (M.B.); alberto.dalmolin@med.uniupo.it (A.D.M.); 2 A.O.U. Maggiore della Carità Hospital, Corso Mazzini n. 18, 28100 Novara, Italy; antonella.molon@maggioreosp.novara.it (A.M.); gerardo.dinardo@maggioreosp.novara.it (G.D.N.); cristina.torgano@maggioreosp.novara.it (C.T.)

**Keywords:** fundamental nursing care, missed nursing care, oral health, mouth care, cross-sectional study

## Abstract

Background: The Fundamentals of Care framework emphasizes a patient-centered approach that prioritizes the nurse–patient relationship and care environment to meet patients’ basic needs, including oral hygiene. Recognized as crucial for preventing systemic health problems, oral care neglect is a global concern. Studies identify missed oral care as a widespread issue, contributing to significant patient safety risks. This study aimed at measuring missed nursing care occurrence in a Northern Italian university hospital, exploring the association between missed oral care with nursing staff characteristics and oral care policies. Methods: A single-center cross-sectional study was conducted according to the STROBE guidelines. Data collection was performed in May 2022 using the MISSCARE survey, a self-administered questionnaire sent by email to 473 nurses from all inpatient units. The degree of implementation of oral care policies was obtained by accessing the Facility Score Sheet data at the department level. Descriptive statistics, chi-square tests, and Fisher tests were conducted using SAS 9.4 and R software. Results: Providing oral care was the third-most missed nursing care after rotating patients every two hours and walking them three times daily. The reasons for missed care included resource shortage and high patient turnover. The Facility Score Sheet data showed a low adoption of oral care policies. No significant associations were found between missed oral care and both nurses’ characteristics and oral care policy at the department level. Conclusions: The study confirms highly missed oral care in acute care settings, emphasizing the urgent need for systemic changes via an evidence-based oral care policy and practice implementation. This study was prospectively registered under protocol 293 CE 050/2022 (8 Aril 2022).

## 1. Introduction

Fundamental care in nursing encompasses a range of activities and approaches focusing on the patient’s essential physical and psychosocial needs [1]. Kitson’s Fundamentals of Care (FOC) framework emphasizes the necessity of a patient-centered approach and the commitment to patient well-being, integrating three key dimensions: the nurse–patient interaction, the comprehensive assessment of care needs, and the healthcare setting where care is provided. The framework is visually represented using concentric circles. At the center is the nurse–patient relationship; the subsequent circles represent the increasing complexity and broader context of healthcare delivery, including the immediate care environment and the wider healthcare system [2].

A key to the effective implementation of Kitson’s FOC framework is a shared understanding of fundamental care’s essential elements. This shared understanding is well-articulated in the Delphi study by Feo et al. [3], which identifies crucial components of fundamental care such as nutrition, elimination, and personal hygiene, including oral care.

Oral diseases, affecting nearly half of the global population (approximately 3.5 billion people), represent a significant health burden, particularly in low- and middle-income countries where three-quarters of affected individuals reside. The major oral health issues encompass dental caries (also known as tooth decay), advanced gum disease, tooth loss, and oral cancers. Globally, untreated dental caries stands out as the predominant condition, impacting approximately 2.5 billion individuals. Severe gum disease, a significant contributor to complete tooth loss, is estimated to afflict around 1 billion people worldwide. The World Health Organization reports significant disparities in oral health treatment accessibility, disproportionately affecting vulnerable and disadvantaged individuals [4]. In the past three decades, the incidence of oral diseases has alarmingly increased by one billion cases, underlying a widespread lack of access to preventive and therapeutic oral health services [5].

There is a well-established relationship between oral care and various systemic conditions. Severe gum diseases have been linked to diabetes, cardiovascular disorders, high blood pressure, stroke, dementia, respiratory diseases, preterm birth, and increased mortality [6,7]. Poor oral care can lead to periodontal disease, which is associated with systemic inflammation. This inflammation has been linked to an increased risk of cardiovascular diseases, as it may contribute to the progression of atherosclerosis and other heart-related conditions. In conditions like diabetes, there is a bidirectional relationship between the disease and periodontal issues: poor glycemic control can worsen gum disease, while severe gum disease can negatively affect blood sugar levels, further complicating diabetes management [8]. With regards to acute care setting, Zhao et al. investigated the impact of oral care on ventilator-associated pneumonia (VAP) incidence in critically ill patients on mechanical ventilation. They found that using chlorhexidine mouthwash or gel reduced VAP incidence from 26% to about 18% compared to placebo or the usual care [9].

As previously mentioned, oral care is recognized as a core component of fundamental nursing care, playing a crucial role in health promotion. This includes the prevention and treatment of oral cavity diseases through comprehensive oral examination, meticulous care, and patient education about oral health maintenance [10].

In several studies focusing on Missed Nursing Care (MNC), oral care has been consistently identified as one of the most frequently neglected areas, together with ambulating and turning patients, providing emotional support to patients and families, and teaching them how to self-care [11,12,13,14]. MNC, defined as any aspect of required patient care that is omitted, either in part or in whole, or delayed has been recognized as a key mechanism explaining the extensively demonstrated association between nurse staffing levels and patient outcomes [15,16,17,18].

According to the overview of the review by Chaboyer et al., MNC can be categorized into the following areas: (a) communication and information sharing; (b) self-management and education, including care and discharge planning; (c) fundamental physical care as oral care; and (d) emotional and psychological care, including spiritual support [12]. The reasons for MNC vary across countries; however, the most commonly reported include nursing staff shortages, high admission and discharge volumes during shifts, and unexpected increases in patient acuity, which disrupt workflow predictability [19]. Moreover, several studies have concluded that a better work environment significantly reduces the occurrence of MNC. Promoting effective communication, fostering an ethical climate of care, and ensuring a safe environment that supports good clinical practices can help to prevent or reduce MNC [13].

Nurses play a crucial role in healthcare as gatekeepers, overseeing planning, coordination, provision, and evaluation of care. They provide various interventions prescribed by other healthcare professionals to address illnesses and their complications, alongside initiating their caring intervention to promote health and manage illness responses. As key figures in healthcare delivery, their role is vital for ensuring quality patient outcomes. If the delivery of care from nurses to patients is hindered, patients may miss out on necessary services, leading to incomplete care processes [20].

Extensive global research has highlighted the significant consequences of neglected nursing responsibilities for patient safety and well-being. These adverse outcomes include substandard care; increased mortality rates; reduced patient satisfaction; and a higher incidence of adverse events such as medication errors, falls, pressure ulcers, infections, and hospital readmissions [21,22]. Even in the Italian context, most studies conducted before and during the COVID-19 pandemic for measuring the occurrence of MNC in acute settings have identified oral care among those most frequently omitted or delayed [14,23,24,25].

Understanding the pattern of MNC in an acute care context allows for tailoring interventions to improve nursing practice and address fundamental care needs.

This study aimed at measuring the occurrence of MNC, as perceived by registered nurses (RNs), in a Northern Italian university hospital, with specific interest in oral care, intending to inform future quality improvement interventions. The secondary aims were to elucidate the reasons for MNC and to explore associations between missed oral care and (1) nursing staff characteristics and (2) the degree of good oral care practice implementation at the department level.

## 2. Materials and Methods

### 2.1. Design

This study adopted a cross-sectional, single-center survey design. It was conducted according to the Strengthening the Reporting of Observational Studies in Epidemiology (STROBE) statement [26].

### 2.2. Setting and Participants

This study was conducted in a 640-bed university hospital in Northern Italy. All inpatient units were eligible, and all 437 frontline RNs, employed either full-time or part-time in the eligible units, were invited to participate in the survey. RNs in managerial positions, those not directly involved in patient care, and those assigned to outpatient services were excluded from the study.

To enable possible comparisons across different hospital departments, a sample size was calculated using G*Power 3.1 software. Alpha and beta values were set at 0.05 and 0.20, respectively, to establish the margin of error. For meaningful comparisons, a sample size of 216 RNs was defined based on an anticipated effect size of 0.25 (mean effect) for a 6-group ANOVA encompassing the Medical, Surgical, Emergency, Oncology, Cardiothoracic, and Maternal Departments.

### 2.3. Data Collection

Data collection was performed in May 2022. Before collecting data, all eligible RNs were informed about the study’s aims, the data collection procedure, and the assurance of data confidentiality. The process involved a self-administered questionnaire, which included the Italian MISSCARE survey and its demographic section. The questionnaire was distributed using a web survey method via REDCap (Research Electronic Data Capture) software. Invitation emails were initially sent by the principal investigator to all eligible RNs, followed by a reminder email one month later to those who had not responded. The estimated time for the completion of the survey was 10–15 min [24,27].

The degree of implementation of good oral care practices was obtained by accessing the Facility Score Sheet (FSS) data at the departmental level. The FSS, a tool adopted by nurse managers to assess oral and dental care, allows to track the implementation status of good oral care practices and the development of new policies within healthcare facilities [28].

### 2.4. Survey Tools and Study Variables

#### 2.4.1. MISSCARE Survey—Italian

For the purpose of measuring the occurrence of MNC, the MISSCARE Survey in its Italian validation was taken into account [24,27]. The MISSCARE survey, which has been thoroughly validated and is extensively utilized in many countries, stands as a reliable instrument for measuring MNC. In the Italian-validated version, the internal consistency of the survey was evaluated using Cronbach’s coefficient, resulting in a reliability score of 0.94 [24]. Permission to use the tool was obtained from the authors in June 2021.

The Italian MISSCARE survey consists of two sections: Part A and Part B. Part A includes 24 items, while Part B comprises 17 items. Part A specifically focuses on the elements of MNC, measuring the frequency of nursing care activities that are delayed or omitted, each rated on a 5-point Likert scale. On this scale, a score of ‘5’ indicates that care is ‘always missed’, whereas a score of ‘1’ signifies ‘never missed’. The scale also includes intermediate options such as ‘frequently’, ‘occasionally’, and ‘rarely’ to capture the varying frequency of MNC. On the other hand, Part B explores the reasons for MNC, an inventory of potential reasons why care activities are delayed or missed. It includes 17 items that assess the level of agreement regarding potential causes for MNCs. This section employs a 4-point Likert scale, with ‘4’ denoting a ‘significant reason’, ‘3’ a ‘moderate reason’, ‘2’ a ‘minor reason’, and ‘1’ representing a ‘non-significant reason’ for MNC. Data on both missed care elements and the reasons for such missed care are gathered based on RNs’ perceptions of the occurrence.

The MISSCARE survey comprises a demographic section covering details like age, gender, education, experience, workload, type of working shift, and overtime. The form also included two questions regarding the RNs’ intention to leave their current unit and their job satisfaction.

#### 2.4.2. Facility Score Sheet

The FSS consists of 12 questions rated on a 4-point Likert scale. It encompasses aspects including written policies, continuing education courses, consultation with patients’ dental specialists, oral assessments, plans for oral hygiene care, and the frequency of oral hygiene care. The overall score is the sum of the scores for each item, ranging from 0 to 36 [28]. Higher scores indicate better levels of oral care practices and policy implementation.

#### 2.4.3. Study Variables

The variables considered in this study align with the elements of Kalisch’s conceptual framework: (1) antecedents of MNC, (2) MNC, and (3) consequences of MNC [15].

For the antecedents of MNC, the variables measured include nursing staff characteristics (age, gender, education, department of employment, role seniority, ward seniority, shift profile, number of patients cared for per shift, and overtime hours); unit/department characteristics (resource adequacy as perceived by RNs and the FSS related to oral care practices and policy implementation); and reasons for MNC as perceived by RNs.

For the consequences of MNC, the variables measured focus solely on outcomes related to nursing staff, including satisfaction with their current role, satisfaction with being a nurse, satisfaction within their workgroup, and their intention to leave the current unit.

### 2.5. Ethical Consideration

This study received approval from the Official Local Ethic Committee (CE 050/2022, prot. 293, 8 April 2022) and complied with national regulations for handling sensitive and non-sensitive personal data. Detailed information about data management and protection was provided to participants. Data collection was conducted anonymously and aggregated for analysis. To ensure confidentiality, each participant was assigned a unique alphanumeric identification code. All procedures were performed in compliance with relevant laws and institutional guidelines.

### 2.6. Data Analysis

Descriptive statistics were reported for the entire sample, including absolute and percentage frequencies for the categorical variables and means and standard deviations (SD) for the numerical variables.

To analyze the percentage of MNC for each specific element of nursing care (e.g., turning, mouth care, medications on time, etc.), the MISSCARE items—part A were initially categorized as dichotomous variables. Consistent with previous studies by Kalish elements of nursing care were considered missed if reported as ‘always missed’ or ‘frequently missed’ [27]. Conversely, elements of nursing care were considered not missed if marked as ‘occasionally’, ‘rarely’, or ‘never missed’. Parallel to what was done to analyze the frequency of MNC, the reasons for MNC (part B of the tool) were dichotomized into significant reasons and non-significant reasons. Reasons for MNC were considered significant if marked as a ‘significant’ or ‘moderate’ reason.

In addition to the percentage of MNC, the overall mean score of MNC was calculated. This score reflects the average amount of missed care reported on the Likert scale for each item. Higher scores indicate a greater extent of MNC according to RNs’ perceptions. A radar plot was used to enhance the visualization of MNC occurrences, with extreme values highlighting the care most frequently missed. The same analysis was performed regarding part B of the tool—reasons for MNC. Finally, the association between missed oral care and demographic variables was analyzed using the chi-square or Fisher test where appropriate. The demographic variables under consideration were categorized consistently with what has already been documented in previous studies on the MNC topic. Univariate logistic regression models were utilized, with missed oral care as the outcome and various demographic variables as covariates. The results were reported as odds ratios (ORs), along with 95% confidence intervals (CIs).

The FSS for measuring good oral care practice was calculated at the department level, and differences were graphically presented using box plots. To test differences among departments, an ANOVA test was performed, and statistically significant results were reported.

All the analyses were conducted using SAS 9.4 and R software; the statistical threshold was set to 0.05, two-tailed.

## 3. Results

### 3.1. Demographic Characteristics

Out of the 437 RNs invited to participate, 131 (29.98%) returned the questionnaire filled in (Table 1). Surgical and medical departments were the most represented ones (48, 60.31% and 31, 23.66%, respectively).

The RNs were mainly female (106, 80.92%), with an average age of 39.31 years (SD = 11.5), and most were working on rotating shifts (116 respondents, representing 89.23%). Nearly half of the respondents (59, 45.74%) have been working as RNs for more than ten years, and approximately one-third (36, 27.91%) have been employed in the same unit for more than ten years. On average, RNs reported caring for 11.55 patients (SD = 5.75) per shift and accumulating an average of 53.96 overtime hours (SD = 145.53) in the past three months. Most RNs (130, 76.92%) did not express an intention to leave their unit and were satisfied with their roles (96, 73.28%).

### 3.2. Part A—Elements of Missed Care

The study identified the following MNC activities as the most frequently reported by nurses: turning patients every 2 h (56.49%), ambulating patients three times per day or as ordered (52.71%), providing mouth care (28.24%), teaching patients about plans for their care after discharge (27.13%), and educating patients and caregivers (24.43%). Hand washing (3.2%), bedside glucose monitoring as ordered (4.58%), wound care (4.58%), and PRN medication requests acted on within 15 min (5.34%) were the least missed or delayed nursing care, according to the study’s participants.

**Figure 1:**
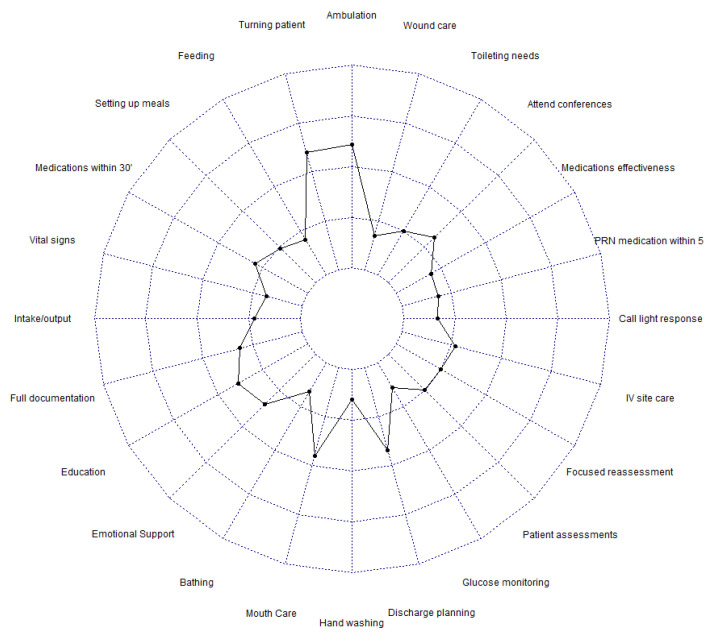
Radar chart representing MNC.

The overall mean score of MNC was calculated, and a visual representation of MNC was provided by a radar chart (Figure 1), where higher scores indicate a greater extent of MNC according to the RNs’ perceptions.

### 3.3. Part B—Reasons for MNC

The study revealed several significant reasons for MNC, including, at the top, an inadequate number of staff (86.26%) and an unexpected number of admissions/discharges throughout the shift (86.26%). An inadequate number of assistive personnel (82.44%), unexpected rise in patient volume and/or acuity in the unit (81.68), and urgent patient situations (79.39%) are the other three most significant reasons identified according to the participants’ viewpoints.

**Figure 2:**
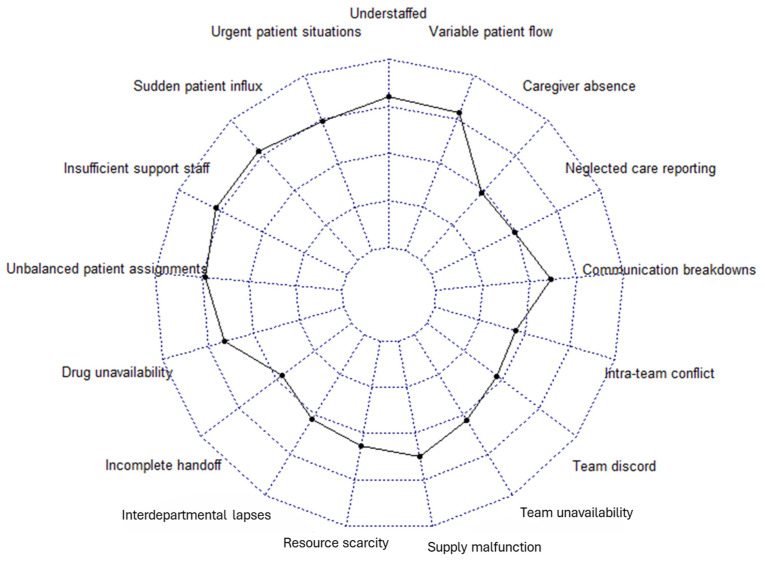
Radar chart representing the reasons for MNC.

Similar to part A of the survey, an overall mean score for the reasons behind MNC was calculated and displayed in the radar chart in Figure 2. Here, higher scores represent more significant reasons for missing care as perceived by the RNs.

### 3.4. Oral Care and Nursing Staff Characteristics

Given that this study served as an initial phase to guide subsequent quality improvement initiatives, with a specific interest in oral care, it focused not only on measuring MNC and identifying their underlying reasons but also explored the association between the occurrence of missed oral care and the characteristics of nursing staff participants, including the hospital department where they are employed.

Missed oral care was reported by almost one-third of RNs (N = 37, 28.24%). No statistically significant association emerged between the RNs’ characteristics and missed oral care reported. The only data close to significance were the department of employment (Table 2). Due to the lack of statistically significant associations, more advanced logistic regression models, such as multivariate or multilevel analyses, were not conducted to avoid overfitting the data or drawing potentially misleading conclusions.

### 3.5. Good Oral Care Practice Implementation

According to the FSS, accessed for each of the involved units, the overall score for good oral care practice and policy implementation was 5.83 (SD 3.849), on a scale ranging from 0 to 36. In 75% of the units, specific oral care policies, continuing education courses, and consultations with specialists were almost absent. Furthermore, in most cases (84.09%), the FSS indicated the lack of an oral hygiene care plan.

**Figure 3:**
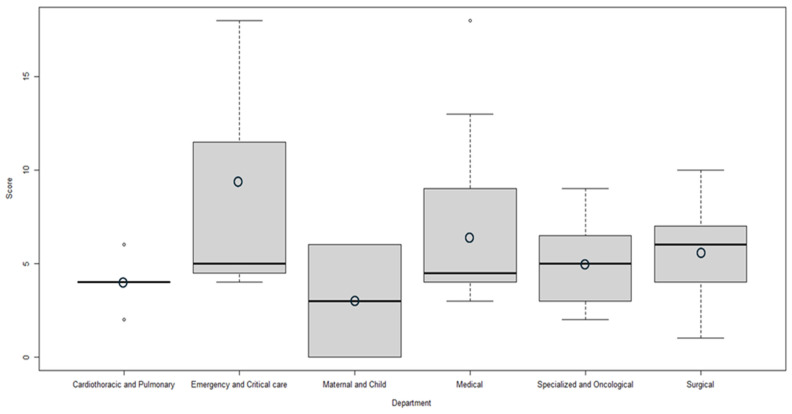
FSS for good oral care practice and policy implementation at the department level.

Figure 3 shows the FSS at the department level, highlighting better scores in the Emergency and Critical Care Department (mean = 9; SD = 7.81) and lower scores in the Maternal and Child Department (mean = 3, SD = 4.24). The results showed an overall low adoption of good oral care policies and practices, but it was not possible to identify statistically significant differences among the departments (*p* = 0.3679).

## 4. Discussion

This study found that the three most frequently reported MNCs were rotating patients every two hours, walking patients three times daily or as prescribed, and providing oral care. On the contrary, the least missed or delayed care included bedside glucose tests, wound care, and timely response to PRN medication requests. This pattern, especially regarding oral care, aligns with the results from previous Italian [24,25] and international studies [12,29,30].

The data of the present study were collected right after the last wave of COVID-19, as Italy extended its state of emergency until 31 March 2022 [31]. According to the available literature on MNC, the hierarchy among the different elements of nursing care was the same both before and during the COVID-19 pandemic, and oral care was constantly reported among the most omitted [32,33]. This stability in the prioritization pattern could be due to several factors: the RNs’ reliance on standardized decision-making processes, a kind of implicit reasoning where certain activities are prioritized over others; the limitations of existing tools to measure MNC, which were not designed for pandemic conditions; and the possibility that RNs have historically made decisions under resource scarcity, suggesting continuity in their prioritization patterns [34]. Consequently, during the pandemic, RNs might have adhered to their established practices, lacking the time for reflecting on or receiving the necessary training to navigate the new challenges effectively.

Concerning the reasons for MNC measured in this study, the three most reported were the lack of human resources, high patient turnover, and unexpected rise in patients’ volume and acuity. These results also overlapped with previous research on MNC conducted both before and after the COVID-19 pandemic, suggesting that RNs’ decision-making regarding care prioritization may be influenced by persisting resource constraints and overwhelming workloads [24,33,35]. Confirming this hypothesis, the nurse-to-patient ratio reported in this study was 1:11.5, higher than the recommended ratio of 1:6 for medical and surgical units in Italian acute care settings [36]. As the global study RN4CAST highlighted, an increase in an RN workload by even just one patient can compromise the quality of healthcare outcomes other than contributing to the occurrence of MNC [16].

Missed oral care in hospitalized patients can lead to significant adverse effects, including the deterioration of oral health, which is associated with an increased risk of hospital-acquired infections and reduced quality of life. Maintaining a clean and healthy mouth enhances the overall well-being by supporting fluid and nutritional intake and facilitating clear speech and communication [37,38]. Despite the critical importance of oral care, the literature currently includes only one preliminary report addressing the issue of missed oral care [39], with a lack of research exploring the association with nursing staff characteristics.

As the present study was the initial step towards guiding future quality improvement in oral care practice at Novara University Hospital, the authors were interested in understanding the association between missed oral care and RNs’ characteristics, with a particular emphasis on their departmental affiliations. This focus arose from the hypothesis that the organizational context of departments, and their level of implementation of good oral care practices, could have an impact on the amount of missed oral care. No significant association emerged from the data collected, probably due to the low number of RNs responding to the survey and the failure to achieve the sample size necessary to observe statistically significant differences.

The data on the level of oral care practice and policy, despite unable to catch statistically significant differences among departments, showed an overall low adoption of oral care policy, highlighted by the absence of a continuing education program on oral health and a notable lack of oral hygiene care plans in patient records. The report by Charalambous et al. [39] showed that barriers to effective oral care in acute care settings include patient characteristics and lack of knowledge, attitudes, and skills among nurses, as well as challenges in the work environment, such as heavy workloads and insufficient resources. As facilitators to enhance oral care, the authors emphasized the importance of educating both patients and nurses, establishing formal oral care protocols, and the crucial role of nurse managers in supporting care standards adoption. The findings suggest a need for systemic changes, including continuous education and protocol development, to improve oral care practices in hospital settings. Concerning barriers to good oral care practice implementation, another study revealed that a significant majority of nurses faced obstacles in providing oral care, including lack of equipment, absence of guidelines, staff shortages, time constraints, inadequate knowledge, poor supervision, and high workload [40]. These barriers suggest a need for wide-ranging improvements at the individual, organizational, and governmental levels to enhance oral care practices in hospitals.

Within the framework of evidence-based healthcare, enhancing the provision of fundamental nursing care necessitates initiatives to develop solid evidence, designing and testing rigorous interventions to assess the impact and effectiveness of fundamental care delivery, thereby facilitating their expansion both within and across healthcare systems [41,42]. Despite the explored association’s failure to confirm a relation between missed oral care as perceived by RNs and the extent of good oral care practice implementation, both the high occurrence of missed oral care reported and the poor adoption of oral care policies highlighted a concern for patient safety and laid the groundwork for future quality improvement initiatives.

Moreover, although the hierarchy among the different elements of MNC appears to have remained consistent both before and during the COVID-19 pandemic, with oral care consistently reported as one of the most frequently omitted tasks, this stability in prioritization patterns may reflect the limitations of existing tools for measuring MNC under pandemic conditions. Investigating whether nursing tasks like oral care were disproportionately deprioritized only during the pandemic, with a subsequent return to pre-pandemic norms, or if this de-prioritization has persisted and become an established practice, potentially leading to adverse patient outcomes, remains a critical area for future research.

The present study has several limitations. Firstly, the survey response rate was lower than expected, resulting in a final sample size of 131 nurses, below the initially calculated target of 216 participants. This may have introduced response bias and limited the generalizability of the findings to the wider nursing population. However, it is important to emphasize that the primary aim of the study was descriptive: to measure the occurrence of MNC and identify its underlying reasons. While the reduced sample size may have impacted the precision of some estimates or the detection of smaller trends, it does not preclude the study from offering meaningful insights into the prevalence of MNC and its reasons. Nevertheless, the lower-than-expected response rate has reduced the statistical power to detect significant associations and perform subgroup analyses.

Research conducted during the COVID-19 pandemic observed significantly lower response rates (15.5–17.9%) compared to the pre-pandemic era (>50%) [24,25]. This decline can be attributed to a variety of reasons, such as a diminished emphasis on completing surveys and the fatigue experienced by nursing staff overwhelmed by the sheer volume of questionnaires, many of which were perceived as having questionable value [34].

Secondly, the use of self-reported surveys to measure MNC and its underlying reasons introduces a possible risk of bias and might not fully capture the extent of the phenomenon. This is particularly relevant considering that, during the pandemic, RNs adopted behaviors or practices that current tools for assessing MNC could be unable to identify.

Finally, this study did not include post hoc validation with imputation techniques or additional exploratory analyses to assess the robustness of the findings. While such approaches could provide further insights, they were beyond the scope of the study’s aims. Future research could explore the application of such techniques to strengthen the robustness of the findings in similar contexts.

## 5. Conclusions

The compromise in delivering fundamental care negatively impacts patients, their families, healthcare personnel, and the healthcare system as a whole. The data on MNC reveal that shortcomings in basic care provision are not limited to any single country or health infrastructure; rather, the failure to administer fundamental nursing care presents a global challenge.

This study found that oral care was reported as missed by nearly one-third of participating nurses (28.24%), making it one of the most frequently omitted aspects of fundamental care in acute settings. Among the reasons identified for MNC were an inadequate number of staff, unexpected fluctuations in admissions or discharges during shifts, and unexpected increases in patient volume or acuity. Additionally, departmental variations in the implementation of oral care practices has emerged as a potential factor influencing the prevalence of MNC.

These findings highlight the urgent need for a systematic approach to address this issue, particularly through the implementation of evidence-based oral care policies and practices tailored to the unique demands of acute care settings.

## Figures and Tables

**Table 1:** Sample characteristics.

Variables	N = 131
**Age, µ (SD)**	39.31 (11.55)
**Gender, n (%)**	
Female	106 (80.92)
Male	
**Role seniority, n (%)**	
Less than 2 years	20 (15.50)
From 2 to 10 years	50 (38.76)
Over 10 years	59 (45.74)
Missing	2 (1.53)
**Ward seniority, n (%)**	
Less than 2 years	41 (31.78)
From 2 to 5 years	37 (28.68)
From 5 to 10 years	15 (11.63)
Over 10 years	36 (27.91)
Missing	2 (1.53)
**Shift profile, n (%)**	
Rotating shift	116 (89.23)
Day shift only	14 (10.77)
Missing	1 (0.76)
**Intention to leave the unit, n (%)**	
Yes, within the next 6–12 months	30 (23.08)
No intention	100 (76.92)
Missing	1 (0.76)
**Satisfaction with the role covered** **, n (%)**	
Very satisfied/satisfied	79 (60.77)
Neither satisfied nor dissatisfied	29 (22.31)
Dissatisfied	18 (13.85)
Very dissatisfied	4 (3.08)
Missing	1 (0.76)
**Satisfaction in being a nurse, n (%)**	
Very satisfied	53 (40.46)
Satisfied	43 (32.82)
Neither satisfied nor dissatisfied	20 (15.27)
Dissatisfied	9 (6.87)
Very dissatisfied	6 (4.58)
**Satisfaction within the group, n (%)**	
Very satisfied	20 (15.38)
Satisfied	58 (44.62)
Neither satisfied nor dissatisfied	35 (26.92)
Dissatisfied	11 (8.46)
Very dissatisfied	6 (4.62)
Missing	1 (0.76)
**Resources considered adequate, n (%)**	
≥50% of the time	89 (67.94)
<50% of the time	42 (32.06)
**Patients cared for per shift, µ (SD)**	11.55 (5.75)
**Overtime hours, µ (SD)**	53.96 (145.53)

Legend: μ = average; SD = standard deviation.

**Table 2:** Association between missed oral care and nursing staff characteristics.

	Oral Care Missed N = 37	Oral Care Not Missed N = 94	*p*	OR [95% CI]
**Age**				
<30	11	26	0.8127	Ref
30+	26	68		0.90 [0.39; 2.09]
**Sex**				
Female	31	75	0.6003	Ref
Male	6	19		0.76 [0.28; 2.10]
**Educational level**				
Diploma or bachelor’s degree	32	75	0.3722	Ref
Master’s degree/Specialization	5	19		0.62 [0.21; 1.80]
**Role seniority** (missing = 2)				
<5 years	12	33	0.8182	Ref
5+ years	24	60		1.10 [0.49; 2.48]
**Ward Seniority** (missing = 2)				
<5 years	23	55	0.8026	Ref
5+ years	14	37		0.91 [0.41; 1.98]
**Intention to leave the unit** (missing = 1)				
Yes, within the next 6–12 months	11	19	0.1696	1.74 [0.73; 4.14]
No intention	25	75		Ref
**Role satisfaction** (missing = 1)				
Very satisfied/satisfied	21	58	0.3539	Ref
Neither satisfied nor dissatisfied	7	22		0.88 [0.33; 2.36]
Dissatisfied/Very dissatisfied	9	13		1.91 [0.71; 5.13]
**Satisfaction in being a nurse**				
Very satisfied/satisfied	28	68	0.6368	ref
Neither satisfied nor dissatisfied	4	16		0.61 [0.19; 1.98]
Dissatisfied/Very dissatisfied	5	10		1.12 [0.38; 3.87]
**Satisfaction within the group** (missing = 1)				
Very satisfied/satisfied	23	55	0.8853	ref
Neither satisfied nor dissatisfied	10	25		0.96 [0.40; 2.31]
Dissatisfied/Very dissatisfied	4	13		0.74 [0.22; 2.50]
**Departments of employment**				
Medical Department	11	20	0.0578	Ref
Surgical Department	16	32		0.91 [0.35; 2.35]
Oncology Department	1	15		0.12 [0.01; 1.05]
Emergency Department	2	17		0.21 [0.04; 1.10]
Cardiothoracic Department	5	7		1.30 [0.33; 5.08]
Maternal Department	2	3		1.21 [0.18; 8.39]

## Data Availability

The datasets used and analyzed during the current study are available from the corresponding author upon reasonable request.

## Guidelines and Standards Statement

The report was conducted according to the Strengthening the Reporting of Observational Studies in Epidemiology (STROBE) statement [23].
